# Rapid and Efficient Identification of *Caenorhabditis elegans* Legacy Mutations Using Hawaiian SNP-Based Mapping and Whole-Genome Sequencing

**DOI:** 10.1534/g3.115.017038

**Published:** 2015-03-04

**Authors:** Aimee Jaramillo-Lambert, Abigail S. Fuchsman, Amy S. Fabritius, Harold E. Smith, Andy Golden

**Affiliations:** National Institute of Diabetes and Digestive and Kidney Diseases, National Institutes of Health, Bethesda, Maryland 20892

**Keywords:** whole-genome sequencing, Hawaiian SNP mapping, CRISPR/Cas9, essential genes, *cdc-25*

## Abstract

The production of viable embryos requires the coordination of many cellular processes, including protein synthesis, cytoskeletal reorganization, establishment of polarity, cell migration, cell division, and in *Caenorhabditis elegans*, eggshell formation. Defects in any of these processes can lead to embryonic lethality. We examined six temperature-sensitive mutants as well as one nonconditional mutant that were previously identified in genetic screens as either embryonic lethal (maternal-effect or zygotic lethal) or eggshell defective. The responsible molecular lesion for each had never been determined. After confirmation of temperature sensitivity and lethality, we performed whole-genome sequencing using a single-nucleotide polymorphism mapping strategy to pinpoint the molecular lesions. Gene candidates were confirmed by RNA interference phenocopy and/or complementation tests and one mutant was further validated by CRISPR (Clustered Regularly Interspaced Short Palidromic Repeats)/Cas9 gene editing. This approach identified new alleles of several genes that had only been previously studied by RNA interference depletion. Our identification of temperature-sensitive alleles for all of these essential genes provides an extremely useful tool for further investigation for the *C. elegans* community, such as the ability to address mutant phenotypes at various developmental stages and the ability to carry out suppressor/enhancer screens to identify other genes that function in a specific cellular process.

Early studies using the model organism *Caenorhabditis elegans* sought to elucidate complex biological processes by modeling genetic studies performed in bacteriophage, *E. coli*, yeast, and *Drosophila* (Edgar *et al.* 1964; Edgar and Lielausis 1964; Wright 1970; Wright and Shaw 1970; Hartwell *et al.* 1974; Hereford and Hartwell 1974). The reasoning behind this approach was to use mutagenesis to accumulate developmental intermediates that could be classified and ordered on the basis of their phenotypes. *C. elegans* is a particularly useful model for studying developmental processes of metazoans because of its small size, amenable genetics, anatomical simplicity, invariant cell lineage, and transparent body and embryos. The early studies using *C. elegans* applied this strategy to analyze nervous system development, behavior, and embryonic/zygotic development (Brenner 1973; Hirsh and Vanderslice 1976; Miwa *et al.* 1980; Schierenberg *et al.* 1980; Wood *et al.* 1980; Cassada *et al.* 1981). With the goal of reaching genetic saturation to provide a clear picture of a given developmental pathway, these studies generated about 600 embryonic (emb)/zygotic (zyg) lethal mutants and 54 maternal-effect lethal (mel) mutants. Through linkage analysis and complementation tests, this large data set of mutants was distilled down to approximately 60 *emb/zyg* genes and 32 *mel* genes (Hirsh and Vanderslice 1976; Miwa *et al.* 1980; Schierenberg *et al.* 1980; Wood *et al.* 1980; Cassada *et al.* 1981; Kemphues *et al.* 1988). Since these early studies, more screens, both conditional and nonconditional, have been conducted, some in hopes of saturating the genome for essential genes (Rose and Baillie 1980; Meneely and Herman 1981; Sigurdson *et al.* 1984; Johnsen and Baillie 1991; Johnsen *et al.* 2000) and thus the number of genes that can be mutated to reveal essential embryonic phenotypes is now much greater. Mutants identified in these early screens are referred to as legacy mutants.

Several of these early screens focused on isolating temperature-sensitive mutations of developmental processes (Hirsh and Vanderslice 1976; Miwa *et al.* 1980; Schierenberg *et al.* 1980; Wood *et al.* 1980; Cassada *et al.* 1981; Kemphues *et al.* 1988). In these original screens, temperature-sensitive mutants made it possible to recover and maintain potentially lethal mutations at the permissive temperature and, as a tool, to understand the temporal control of gene function. Another advantage of temperature-sensitive mutants is that temperature-shift experiments could be performed at various times during development revealing new functions for a particular gene during different developmental stages. This is a big advantage over current methods of gene depletion such as RNA interference (RNAi), which usually only uncovers the earliest phenotypes. Temperature-sensitive mutants also are particularly useful in genetic suppressor screens. Suppressor screens allow for an unbiased approach in the identification of second-site extragenic mutations that ameliorate the phenotype of the original mutation. The use of temperature-sensitive alleles has the advantage of being able to perform mutagenesis on homozygous lines of mutations that are otherwise lethal.

The molecular identities of several of the legacy mutants have been determined through traditional cloning methods. However, these methods often are difficult and time-consuming, leaving the identities of hundreds of the legacy mutant alleles unknown. Recent advances in next-generation, whole-genome sequencing (WGS) has made it a rapid and cost-effective technique for the identification of molecular lesions causing a given phenotype (Minevich *et al.* 2012). Doitsidou *et al.* (2010) developed a one step protocol that combines WGS and single-nucleotide polymorphism (SNP) mapping that further eases mutation identification in *C. elegans*. This method backcrosses the mutant of interest (normally generated in the N2 Bristol isolate) to the polymorphic Hawaiian isolate of *C. elegans* generating recombinants that are sequenced in a single pool. The genomic region linked to the mutation of interest is identified as the region with a decrease in Hawaiian SNPs. This region is then analyzed for molecular lesions.

With the intention of identifying additional reagents that could help expand our knowledge of different aspects of embryonic development including meiotic chromosome segregation and eggshell formation, we performed a literature search for legacy mutants that were previously identified in genetic screens and were preliminarily characterized as temperature sensitive. Of the more than 500 temperature sensitive mutants isolated in early screens, we focused on mutants that were characterized as embryonic lethal or reported to have eggshell defects (osmotic sensitivity, irregular shape, and unshelled or thinly shelled embryos). We chose 12 mutants from publications at least 25 yr old in which the molecular lesion producing the phenotype had not been determined. Of those 12 legacy mutants, only six fit the criteria of being highly penetrant temperature-sensitive mutants. We chose an additional mutant, *mel-2(it20)*, that was not temperature-sensitive but fit the criteria of being a legacy mutant and maternal-effect embryonic lethal. These seven were subjected to WGS/SNP mapping. The molecular lesion causing the phenotype of each mutant was confirmed by RNAi phenocopy and/or by complementation tests. Our analysis revealed that all seven of these legacy alleles bore mutations in genes that had only been studied previously in nonconditional mutants or by RNAi (*tyms-1*, *chaf-1*, *tofu-6*, *fasn-1*, *mus-101*, and *cdc-25.2*). Two of the temperature-sensitive mutations [*zyg-2(b10ts)* and *emb-10(k12ts*)], thought to define distinct genes, were shown to be allelic; both had mutations in the *mus-101* gene. The identification of *emb-29(g52ts)* as an allele of *cdc-25.2* was further confirmed by CRISPR/Cas9 gene editing where we rescued the mutation and recreated the temperature-sensitive phenotype by editing the single mutation in a wild-type background. We have demonstrated the ease and utility of one-step WGS/SNP mapping to identify legacy mutations. The strategies presented here could be applied to any temperature-sensitive mutant that remains to be identified molecularly.

## Materials and Methods

### *C. elegans* strains and culture

The following strains were used: N2 (Bristol), GG36: *emb-6(g36)* I, MJ65: *emb-6(hc65)* I, GG43: *emb-14(g43)* I, GG20: *emb-17(g20)* I, MJ63: *emb-10(k12)* I, DH10: *zyg-2(b10)* I, GG52: *emb-29(g52)* V, YHS25: *cdc-25.2(ok597∆)* (V)*/nT1[qIs51]* (IV;V), VC2225: *npp-6(ok2821∆)* (I)*/hT2[bli-4(e937) let-?(q782) qIs48(Pmyo-2*::*gfp*; *Ppes-10*::*gfp*; *Pges-1*::*gfp)]* (I; III), CV87: *syp-4(tm2713∆)* (I)*/hT2[bli-4(e937) let-?(q782) qIs48]* (I;III), VC3196: *smgl-1(ok2423∆)* (I)*/hT2[bli-4(e937) let-?(q782) qIs48]* (I;III), VC1878: *lpd-3(ok2138∆)* I, VC3150: *ekl-1(ok1197∆)* (I)*/hT2[bli-4(e937) let-?(q782) qIs48]* (I;III), MT20434: *chaf-1(n5453∆)* (I)*/hT2[bli-4(e937) let-?(q782) qIs48]* (I;III), CB4856: wild-type Hawaiian isolate, KK359: *mel-2(it20) unc-4(e120)/mnC1 [dpy-10(e128) unc-52(444)]* II, QA137: *tofu-6(yt2)* II; *ytEx100*, AG247: *tyms-1(tm2429∆)* (I)*/hT2 [bli-4(e937) let-?(q782) qIs48]* (I;III), AG248: *mus-101(tm1761∆)* (I)*/hT2 [bli-4(e937) let-?(q782) qIs48]* (I;III). All tm deletion alleles were obtained from the National Bioresource Project as heterozygotes and were balanced with hT2. The presence of the deletion allele was monitored by polymerase chain reaction. All legacy, ok deletion, and tm deletion alleles are available from the *Caenorhabditis* Genetics Center.

### Quantification of embryonic viability

Single L4 hermaphrodites of the indicated genotype were placed on individual plates at 15°, 20°, or 24°. Adult worms were transferred to new plates daily until the sperm was exhausted. Percent hatching is calculated as the number of hatched larvae divided by the total number of eggs laid. For all quantifications, the number of broods scored is indicated in the figure legends.

### RNAi phenocopy and complementation tests

#### RNAi phenocopy:

Gene candidates were obtained from either the Ahringer RNAi feeding library or the OpenBiosystems RNAi feeding library (Timmons *et al.* 2001; Kamath and Ahringer 2003). Bacteria expressing double-stranded RNA were seeded onto MYOB plates containing 25 μg/mL carbenicillin and 1 mM IPTG and allowed to grow at 22° for 48 hr. L4 hermaphrodites were fed bacteria expressing double-stranded RNA specific to a *C. elegans* gene (as indicated in text and figures) and scored for the number of dead embryos *vs.* live progeny. Bacteria expressing *smd-1* and *cdk-1* were used as negative and positive controls respectively.

#### Complementation tests:

Homozygous (or balanced heterozygotes) L4 hermaphrodites of mutant X were mated to homozygous L4 males of mutant Y at 15°. L4 cross progeny were picked to individual plates, incubated at 24°, and examined for embryonic viability as described previously.

*mel-2(it20)* rescue: *mel-2(it20) unc-4(e120)/mnC1* hermaphrodites were mated to N2 males. *mel-2(it20) unc-4(e120)*/++ heterozygous males were then mated to *tofu-6(yt2)* hermaphrodites carrying the *ytEx100* [marked with dominant *rol-6(su1006)*] rescuing transgene. F1 rollers were picked and their RolUnc progeny scored for hatched progeny [*mel-2(it20) unc-4(e120)*; *ytEx100*].

### Whole-Genome Sequencing

Identification of the molecular lesion responsible for each mutant was determined using a SNP-based mapping strategy combined with WGS (Doitsidou *et al.* 2010). Hermaphrodites from mutant strains were crossed with Hawaiian males (strain CB4856). Approximately 200 F2 progeny were singled to fresh 3.5-cm plates and shifted to 24° for 16 hr to reveal the mutant phenotype. The plates were returned to 15° and allowed to produce progeny for one to two generations. The animals from 20 to 50 mutant plates were then pooled and genomic DNA isolated and sheared by sonication. Libraries were prepared using TruSeq reagents and sequenced with a HiSequation 2500 (Illumina, San Diego, CA). Single-read 50-bp sequencing yielded a minimum of 22-fold genome coverage for each library. Variants were identified using a pipeline of BFAST for alignment (Homer *et al.* 2009), SAMtools for variant calling (Li *et al.* 2009), and ANNOVAR for annotation (Wang *et al.* 2010) with *C. elegans* reference genome version WS220 (www.wormbase.org). Hawaiian SNP density was plotted against chromosome position using R (R Development Core Team 2014), and the mapping interval delimited by the absence of Hawaiian SNPs. Mutations within the mapping interval were filtered to remove variants derived from the Hawaiian and pre-mutagenesis strain backgrounds. Candidate genes were defined by homozygous (>85% variant call), nonsynonymous mutations.

### CRISPR/Cas9-mediated single-nucleotide rescue of *emb-29(g52ts)*

Rescue of the cytosine 716 to thymine mutation (codon 239) in *cdc-25.2* in *emb-29(g52ts)* was performed by editing the single nucleotide 716 from T to a wild-type C using the CRISPR/Cas9 technology. An additional silent nucleotide change was made to remove the PAM Cas9 target site to prevent possible recutting of the rescue oligo or the target site (Kim *et al.* 2014). This additional change created an *Eae*I recognition site (underlined to follow), which was used to distinguish the rescued line from N2. *cdc-25.2* was targeted for Cas9 cleavage using the guide sequence (ATGTCTCTCAATGTTTCGG) in pDD162 (Dickinson *et al.* 2013), targeting the non-coding strand. *emb-29(g52ts)* young adults were injected with plasmids containing a dominant *rol-6* (pRF4, a marker for successful injection), the guide RNA (50 ng/μL), and a rescue oligonucleotide (ATGGGTAGCGGCAATCAATGAGAATATACTTGTCATCAAATTCTTTTTCA**G**AGAGTCGGAAAAAGATGTCTCTCAATGTTTCGGC**C**GTGATTCTTCTGAAGCCTTTCGAGGATTCCTTTGCAACAGTTTTGAGGTGG, IDT Coralville, IA; 30 ng/μL). The rescue oligonucleotide contained the nucleotide changes of interest (in bold) and an additional 50 nucleotides of perfect homology on both sides flanking nucleotide 716 and the PAM site (120 ng/μL) (Paix *et al.* 2014; Zhao *et al.* 2014).

Eight *emb-29(g52ts)* young adults were injected, placed on a single MYOB plate with OP50 bacteria, and kept at 15° for recovery. After 24 hr, worms were transferred two per fresh plate and allowed to continue laying embryos. All four plates segregated roller F1 progeny. F1 rollers were singled from each of the four plates and their progeny (F2s) were shifted to 24° after hatching. In addition, 100 F2 L1 non-roller worms from each of the four plates containing the injected worms were shifted to 24°. Plates were checked for live F3 progeny. Worms from plates with live progeny were sequenced to confirm the two-nucleotide changes (Macrogen USA, Rockville, MD). These lines were given the allele designation *av38*.

### CRISPR/Cas9-mediated single-nucleotide recreation of *emb-29(g52ts)*

N2 young adult worms were injected as stated previously with the same target plasmid (50 ng/μL) in addition to a repair oligonucleotide to mutate *cdc-25.2* nucleotide 716 from a C (wild-type) to a T (ATGGGTAGCGGCAATCAATGAGAATATACTTGTCATCAAATTCTTTTTCA**A**AGAGTCGGAAAAAGATGTCTCTCAATGTTTCGGC**C**GTGATTCTTCTGAAGCCTTTCGAGGATTCCTTTGCAACAGTTTTGAGGTGG IDT, Coralville, IA; 20 ng/μl) and to mutate the PAM aforementioned site (nucleotide changes in bold, *Eae*I site underlined). We coinjected with a *dpy-10* target sequence in pDD162 (25 ng/μL) and a rescue oligonucleotide (20 ng/μL) for co-conversion screening purposes (Arribere *et al.* 2014). Seven N2 worms were successfully injected and kept at 15°. Thirty-five F1 rollers were singled and allowed to lay embryos. Of the F1 rollers, eight contained the C681→G silent mutation in the PAM site by restriction digest (confirmed by sequencing), but only one of those eight had the C716→T edit when sequenced. We believe the greater frequency of the silent mutation may be attributable to the Cas9 cut site being only one nucleotide away from the silent mutation, whereas the C716→T site was 34 bases away. To obtain multiple lines, two more N2 young adults were injected with the same constructs as described previously and 34 roller F1s were singled at 15°. Twelve F2 L4 larvae from each of the 34 mothers were singled and shifted up to 24°. After laying embryos, F2s were returned to the permissive temperature (15°) for recovery. After 24 hr, plates were examined for hatching embryos at 24°. In two of the 34 lines ~25% of the animals had a temperature-sensitive embryonic lethal phenotype. Homozygous non-*dpy-10* embryonic lethal lines were recovered from these lines. Sequencing confirmed the C716→T change and both the lines also contained the C681→G silent mutation. These lines were given the allele designation *av40*.

## Results

### The selection and characterization of *C. elegans* mutants for WGS

**Figure 1:**
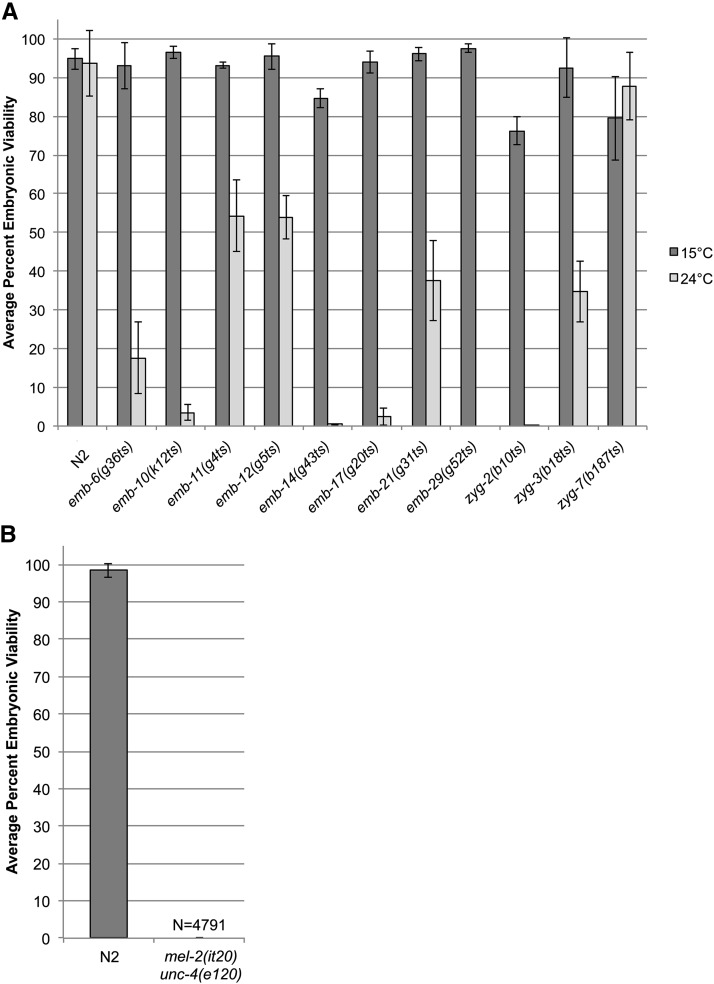
Average percent progeny hatching from mutants evaluated from Table 1. (A) Average percent embryonic viability from N2 and temperature-sensitive mutants. Single L4 hermaphrodites of each mutant strain were placed on individual plates at 15° or 24°. Adult hermaphrodites were transferred daily until the sperm was exhausted. Percent embryonic viability is the number of hatched larvae divided by the total number of embryos laid. At least 19 hermaphrodites were scored for the viability of their progeny for each mutant at 15°. At least 17 hermaphrodites were scored for each mutant at 24°. Error bars represent standard deviation of the average percentage of three individual replicate experiments. (B) Average percent embryonic viability from N2 and *mel-2(it20)*. *mel-2(it20)* animals were marked with *unc-4(e120)* and single L4 Unc hermaphrodites were picked from balanced animals of the genotype *mel-2(it20) unc-4(e120)/mnC1* and placed on individual plates at 20°. Adult hermaphrodites were transferred daily until the sperm was exhausted. Percent embryonic viability was determined as stated above. At least 20 hermaphrodites were scored for the viability of their progeny for each strain. *mel-2(it20) unc-4(e120)* produced zero live progeny from a total of 4791 embryos (N) scored. Error bars represent SD of the average percentage of three individual replicate experiments.

Twelve mutants that had been identified previously in genetic screens but whose molecular identity remained unknown were chosen for analysis and WGS (Table 1). Each mutant was tested for temperature sensitivity and for embryonic lethality by counting the number of dead embryos *vs.* live progeny at both the permissive (15°) and restrictive (24°) temperatures (Figure 1A). One mutant, *mel-2(it20)*, was not temperature sensitive and was maintained by picking balanced heterozygous *mel-2(it20) unc-4(e120)/mnC1* (Figure 1B). These animals were scored for embryonic lethality by picking Unc homozygotes at 20° and counting the number of dead embryos *vs.* live progeny produced. For six mutants, embryonic lethality was temperature-sensitive and strongly penetrant at 24° (Figure 1A). Although all mutants were preliminary characterized as embryonic lethal in the original studies, some mutants showed weak penetrance of the embryonic lethal phenotype at the restrictive temperature and others did not appear lethal at any temperature tested (Figure 1A). The lack of an embryonic lethal phenotype could be due to several reasons such as loss of the original mutations during the 30-plus years of maintaining the strain.

**Table 1:** List of mutants analyzed in this work

Mutant	Preliminary Phenotypic Characterization	References
*emb-6(g36ts)*	Maternally or zygotically required for embryogenesis. Arrest at the 14- to 19-cell stage.	(Schierenberg *et al.* 1980; Miwa *et al.* 1980; Denich *et al.* 1984)
*emb-10(k12ts)*	Arrested embryonic development.	(Herman *et al.* 1980)
*emb-11(g4ts)*	Osmotically sensitive. 94% embryonic arrest during early proliferation.	(Cassada *et al.* 1981)
*emb-12(g5ts)*	Osmotically sensitive. 72% embryonic arrest during early proliferation.	(Cassada *et al.* 1981)
*emb-14(g43ts)*	Osmotically sensitive. 100% arrest.	(Cassada *et al.* 1981)
*emb-17(g20ts)*	Eggs irregular in shape. Arrest at the lima bean stage.	(Cassada *et al.* 1981; Denich *et al.* 1984)
*emb-21(g31ts)*	90% arrested embryonic development at the 26- to 30-cell stage. Eggs round in shape.	(Cassada *et al.* 1981; Denich *et al.* 1984)
*emb-29(g52ts)*	Maternally required for embryogenesis. 100% arrest at the 150- to 200-cell stage. Also affects gonadogenesis.	(Cassada *et al.* 1981; Denich *et al.* 1984)
*zyg-2(b10ts)*	Arrested embryonic development at the 25- to 50-cell stage. Also required for gonadogenesis and male fertility.	(Wood *et al.* 1980; Hecht *et al.* 1982)
*zyg-3(b18ts)*	Maternal and zygotic expression required for embryonic development. Arrest during proliferation. Eggs lyse *in vitro*.	(Wood *et al.* 1980)
*zyg-7(b187ts)*	Maternal and zygotic expression required for embryonic development.	(Wood *et al.* 1980)
*mel-2(it20)*	Maternal-effect lethal. No male rescue.	(Kemphues *et al.* 1988)

List of *emb*, *zyg*, *or mel* mutants analyzed in this paper, description of the mutant phenotypes as described in the original papers, and the references for each mutant.

**Figure 2:**
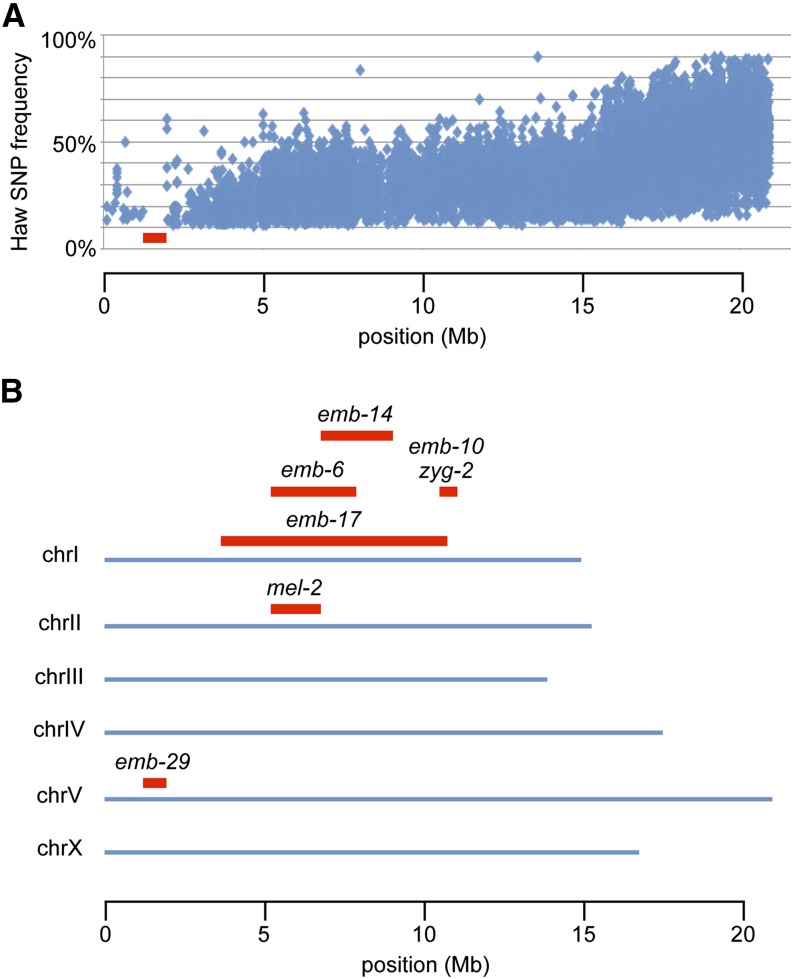
Hawaiian SNP mapping of mutation intervals. (A) Representative plot. Shown is chromosome V from *emb-29*, with the gap in Hawaiian SNPs (red) defining the mapping interval. (B) Summary of Hawaiian SNP mapping results. SNP, single-nucleotide polymorphism.

We chose the six mutants with strong temperature-sensitive embryonic lethality, as well as *mel-2(it20)*, for WGS using the single step SNP mapping strategy of Doitsidou *et al.* (2010) (WGS/SNP mapping) (Doitsidou *et al.* 2010; Wang *et al.* 2014). All seven mutants were recessive. After a single outcross into the Hawaiian strain, CB4856, homozygous F2 embryonic-lethal mutants were reisolated and allowed to grow another one to two generations at the permissive temperature (15°). Pooled populations of F3/F4 animals were harvested for WGS and SNP analysis (see the section *Materials and Methods*). A loss or reduction of Hawaiian SNPs defined the mapping interval containing the mutant allele. A representative graph plotting the Hawaiian SNPs along the chromosome from a population of F2 recombinants is shown in Figure 2A. This strategy produced an average mapping interval of 3.0 Mb (Figure 2B). The mapping interval was then analyzed for missense, nonsense, or splice-site variants in protein coding sequence. An average of five nonsynonymous, candidate mutations were identified per strain with a minimum of two candidates identified for three of the mutants and a maximum of ten candidates identified for one of the mutants (see Tables 2−8). To validate the identity of the molecular lesion responsible for the mutant phenotype, we tested candidates for RNAi phenocopy of the embryonic lethal phenotype or for a failure to complement a deletion allele of the candidate gene. The results of these validation tests are in the following sections.

### emb-6(g36ts) is an allele of tyms-1

*emb-6* had been mapped previously to chromosome I at -0.47 cM. The WGS and SNP-based (WGS/SNP) method of mutant mapping also mapped the molecular lesion to chromosome I and to a 2.8-Mb interval. Analysis of that interval produced eight nonsynonymous sequence variants (Table 2). Of the eight candidates, three were reported to have embryonic lethal phenotypes by RNAi (*tyms-1*, *npp-6*, and *smgl-1*) or by analysis of available deletion alleles [*npp-6(ok2821∆)* and *smgl-1(ok2423∆)*]. To validate the molecular lesion responsible for *emb-6(g36ts)*, we first performed phenocopy experiments by RNAi knock-down of *tyms-1*, *npp-6*, and *smgl-1*. Depletion of *tyms-1* and *npp-6* resulted in embryonic lethality with a penetrance of 89% and 99%, respectively (Table 2). RNAi knock-down of *smgl-1* did not result in embryonic lethality (98% embryonic viability). Because RNAi knock-down of both *tyms-1* and *npp-6* phenocopied the embryonic lethality of the *emb-6(g36ts)* mutant, further validation was required.

**Table 2:** *emb-6(g36ts)* is an allele of *tyms-1*

Strain	Candidate	Mutation	Description	RNAi Phenocopy- Reported on Wormbase	RNAi Phenocopy- This Study	Complementation Test
*emb-6(g36ts)*	***tyms-1***	G->A, Gly42Arg	Thymidylate synthase ortholog	Embryonic lethal	89% embryonic lethal	Fails to complement *tyms-1(tm2429∆)* at 24° (0% hatching, N = 16)
	*npp-6*	G->A, Gly861Arg	Nuclear pore complex	Embryonic lethal	99% embryonic lethal	Complements *npp-6(ok2821∆)* at 24° (94% hatching, N = 14)
	*frm-4*	C->T, Pro241Ser	FERM domain protein	Not embryonic lethal	Not embryonic lethal	NP
	*lpd-2*	G->A, Ala14Thr	Lipid depleted	Not embryonic lethal	NP	NP
	*crml-1*	C->T, Ser66Leu	Capping, ARp2/3, Myosin I linker protein	Not embryonic lethal	Not embryonic lethal	NP
	*T28F4.3*	C->T, Ser116Phe	No information available	Not embryonic lethal	Not embryonic lethal	NP
	*F27D4.6*	G->A, Arg980Lys	No information available	Not embryonic lethal	Not embryonic lethal	NP
	*smgl-1*	C->T, Pro698Ser	SMG-associated and lethal	~50% embryonic lethal	Not embryonic lethal	Complements *smgl-1(ok2423∆)* at 24° (95% hatching, N = 8)

List of gene candidates with genomic DNA sequence variants identified by WGS/SNP mapping of *emb-6(g36ts)*, the missense mutation identified and corresponding amino acid change, a description of the gene function, RNAi phenotypes reported on Wormbase, the percent embryonic lethality by RNAi experiments performed in this study, and the results of complementation tests performed in this study. NP, not performed. N is the number of F1 *trans*-heterozygous animals shifted to 24° for F2 hatching analysis. RNAi, RNA interference; WGS, whole-genome sequencing; SNP, single-nucleotide polymorphism. Bold indicates the gene, when mutated, that is responsible for the embryonic lethal phenotype.

**Figure 3:**
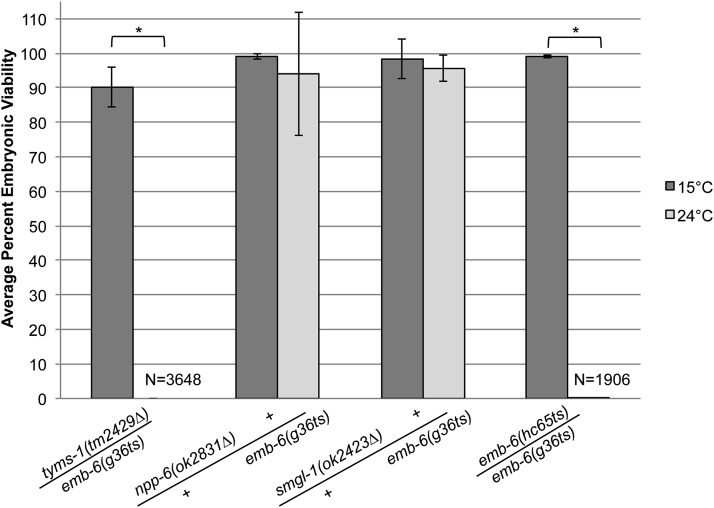
*emb-6(g36ts)* fails to complement *tyms-1(tm2429∆)*. The following complementation tests were performed: *emb-6(g36ts)*; *him-8(e1489)* males crossed with *tyms-1(tm2429∆)/hT2*, *npp-6(ok2831∆)/hT2*, *smgl-1(ok2423∆)/hT2*, and *emb-6(hc65ts)* hermaphrodites. The progeny of single F1 *trans*-heterozygous hermaphrodites were scored for the number of dead embryos *vs.* hatched larvae and the results calculated as the average percent embryonic viability at 15° or 24°C. The broods of at least seven hermaphrodites were scored for each cross at 15°. The broods of at least eight hermaphrodites were scored for each cross at 24°. F1 *trans*-heterozygous hermaphrodites from the *emb-6(g36ts)*; *him-8(e1489)* males crossed with *emb-6(hc65ts)* produced only one hatched larvae out of the 1906 embryos scored. *P < 0.0001. Error bars indicate SD. N = total number of embryos and hatched larvae scored. At least 1100 embryos were scored for each cross.

To determine which of these two mutations is responsible for the *emb-6(g36ts)* embryonic-lethal phenotype, complementation tests were performed. Males of *emb-6(g36ts)*; *him-8(e1489)* were mated to either *npp-6(ok2821∆)/hT2* or *tyms-1(tm2429∆)/hT2* hermaphrodites at 15° (hT2 is a translocation balancer with a pharyngeal GFP insertion). Single non-green F1 cross progeny were picked to individual plates, incubated at 24° and scored for the number of dead embryos *vs.* viable progeny. *emb-6(g36ts)* and *npp-6(ok2821∆)* complemented each other with 94% of the progeny of F1 *trans*-heterozygotes hatching at 24° (Table 2 and Figure 3). We conclude that *npp-6(ok2821∆)* and *emb-6(g36ts)* are not alleles of the same gene. On the other hand *emb-6(g36ts)* and *tyms-1(tm2429∆)* fail to complement (0% hatching, Table 2 and Figure 3) suggesting that *tyms-1* and *emb-6* are the same gene. The *g36ts* allele has a G→A transition (Gly42 to Arg) in *tyms-1*. As further confirmation, we sequenced the *tyms-1* gene in another *emb-6* allele, *hc65ts*, and found a missense mutation in *tyms-1* (C→T), which changes proline 71 to a serine.

Even though *smgl-1 RNAi* did not phenocopy *emb-6*, a deletion mutant allele of *smgl-1* was available from the Caenorhabditis Genetics Center that was embryonic lethal. To further rule out *smgl-1* as a candidate for *emb-6*, we performed complementation tests between *emb-6(g36ts)* and *smgl-1(ok2423∆)*. The *trans*-heterozygotes complemented at 24° (95% hatching, Table 2 and Figure 3) ruling out *smgl-1* as an *emb-6* candidate. Because *tyms-1* is the only candidate that both phenocopied the *emb-6* embryonic lethality and failed to complement, we conclude that *emb-6* is an allele of *tyms-1*, an ortholog of thymidylate synthase. Thymidylate synthase is involved in the pyrimidine biosynthesis pathway and is a major target of 5-fluorouracil, an anticancer drug (Kim *et al.* 2008). The Gly42 to Arg mutation found in *emb-6(g36ts)* and the Pro71 to Ser mutation of *emb-6(hc65ts)* are located in the pyrimidine hydroxymethylase domain.

### emb-17(g20ts) is an allele of chaf-1

Previous mapping of *emb-17(g20ts)* placed it on chromosome I but its position along the chromosome was not determined. WGS/SNP mapping found a reduction in the number of Hawaiian SNPs on chromosome I within a 7.2-Mb interval. This interval contained 10 nonsynonymous sequence variants (Table 3). Of the 10 candidates, four were reported to have some degree of embryonic lethality by RNAi (*rab-10*, *ekl-1*, *smgl-1*, and *chaf-1*). RNAi clones were available for *ekl-1* and *smgl-1*. To determine whether either DNA lesion found in *ekl-1* or *smgl-1* resulted in the same phenotype as *emb-17(g20ts)*, we performed RNAi phenocopy experiments. As stated in the previous section, RNAi knock-down of *smgl-1* did not result in embryonic lethality but RNAi knock-down of *ekl-1* resulted in 42% embryonic lethality (Table 3).

**Table 3:** *emb-17(g20ts)* is an allele of *chaf*-*1*

Strain	Candidate	Mutation	Description	RNAi Phenocopy- Reported on Wormbase	RNAi Phenocopy—This Study	Complementation Test
*emb-17(g20ts)*	*lpd-3*	C->T, Ala467Thr	Lipid depleted	Not embryonic lethal	NP	Complements *lpd-3(ok2138∆)* but some larval lethality in F_2_ at 24° (98% hatching, N = 10).
	*met-1*	C->T, Arg1167Cys	Histone methyltransferase	Not embryonic lethal	NP	NP
	*M04F3.2*	G->T, Glu17stop	No information available	Not embryonic lethal	NP	NP
	*rab-10*	C->T, Ala82Thr	Rab-like GTPase	Embryonic lethal	NP	NP
	*ekl-1*	C->A, Leu582Ile	Enhancer of Ksr-1 Lethality	Range of embryonic lethal	42% embryonic lethal	Complements *ekl-1(ok1197∆)* at 24° (97% hatching, N = 9)
	*rde-2*	G->T, Thr148Asn	RNAi defective	Not embryonic lethal	NP	NP
	*smgl-1*	C->T, Asp1141Asn	SMG-associated and lethal	~50% embryonic lethal	not embryonic lethal	Complements *smgl-1(ok2423∆)* at 24° (87% hatching, N = 5)
	*F55H12.4*	T->C, Lys170Arg	No information available	Not embryonic lethal	NP	NP
	***chaf-1***	C->T, Arg436His	Chromatin Assembly Factor	Embryonic lethal	NP	Fails to complement *chaf-1(n5453∆)* at 24° (4.9% hatching, N = 46)
	*B0205.1*	C->T, Ser268Asn	No information available	Not embryonic lethal	NP	NP

List of genes with genomic DNA sequence variants identified by WGS/SNP mapping of *emb-17(g20ts)*, the missense mutation identified, and corresponding amino acid change, a description of the gene function, RNAi phenotypes reported on Wormbase, the percent embryonic lethality by RNAi experiments performed in this study, and the results of complementation tests performed in this study. NP, not performed. N is the number of F1 *trans*-heterozygous animals shifted to 24°C for F2 hatching analysis. RNAi, RNA interference; WGS, whole-genome sequencing; SNP, single-nucleotide polymorphism. Bold indicates the gene, when mutated, that is responsible for the embryonic lethal phenotype.

**Figure 4:**
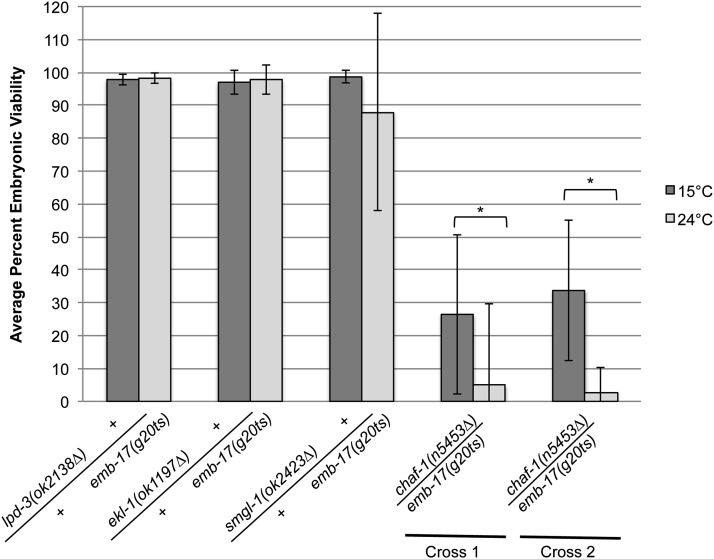
*emb-17(g20ts)* fails to complement *chaf-1(n5453∆)*. Complementation tests were performed by crossing *emb-17(g20ts)*; *him-8(e1489)* males with *lpd-3(ok2138∆)*, *ekl-1(ok1197∆)/hT2*, *smgl-1(ok2423∆)/hT2*, or *chaf-1(n5453∆)/hT2* (Cross 1) hermaphrodites. In addition, *emb-17(g20ts)*; *him-5(e1490)* males were crossed with *chaf-1(n5453∆)/hT2* hermaphrodites (Cross 2). The progeny of single F1 *trans*-heterozygous hermaphrodites were scored for the number of dead embryos *vs.* hatched larvae and the results calculated as the average percent embryonic viability at 15° or 24°. The broods of at least seven F1 hermaphrodites were scored for each cross at 15°. The broods of at least five F1 hermaphrodites were scored for each cross at 24°. *P < 0.0001. Error bars indicate standard deviation. At least 900 embryos were scored for each cross.

Previous analysis of deletion alleles of three of the 10 candidates found them to be sterile [*ekl-1(ok1197∆)*] or embryonic lethal [*smgl-1(ok2423∆)* and *chaf-1(n5453∆*)]. For the remaining candidates, deletion alleles were either not available (*met-1*, *M04F3.2*, *rde-2*, *F55H12.4*, and *B0205.1*) or not characterized as sterile or embryonic lethal (*lpd-3* and *rab-10*). To further validate the mutant candidate responsible for the *emb-17(g20ts)* embryonic lethal phenotype, we performed complementation tests between *emb-17(g20ts)*; *him-8(e1489)* males and hermaphrodites carrying deletion alleles of the gene candidates. Single F1 *trans*-heterozygous cross progeny from these crosses were scored for dead F2 embryos *vs.* viable progeny at both 15° and 24°. *ekl-1(ok1197∆)* and *smgl-1(ok2423∆)* both complemented *emb-17(g20ts)* (97% and 87% hatching of F2 progeny at 24°, Table 3 and Figure 4), suggesting that neither of these genes are allelic to *emb-17(g20ts)*. Although *lpd-3(ok2143∆)* was not characterized to be sterile or embryonic lethal, a deletion allele existed. Therefore, we also performed complementation tests between *emb-17(g20ts)*; *him-8(e1489)* males and *lpd-3(ok2143∆)* hermaphrodites. *lpd-3(ok2143∆)* complemented *emb-17(g20ts)* (98% hatching of F2 progeny at 24°, Table 3 and Figure 4). Only *trans*-heterozygous F1s from *emb-17(g20ts)*; *him-8(e1489)* males crossed with *chaf-1(n5453∆)/hT2* hermaphrodites produced dead embryos (4.9% and 2.8% hatching at 24°, Table 3 and Figure 4). Furthermore, F1 *trans*-heterozygotes were not healthy at the permissive temperature of 15° (Figure 4), suggesting that these two alleles do not make enough active gene product at the permissive temperature to support development. Taken together these data show that *chaf-1(n5453∆)* fails to complement *emb-17(g20ts)*, demonstrating that they are alleles of the same gene that codes for the chromatin assembly factor chromatin assembly factor-1 p150 subunit. Chromatin assembly factor-1 is a three-subunit protein complex (p150, p60, and p48) required for the deposition of histones H3-H4 into nucleosomes during DNA replication (Nakano *et al.* 2011; Smith and Stillman 1989). The *g20ts* allele is a C→T transition that changes Arg436 to His in the p60 binding domain of the p150 subunit.

### emb-10(k12ts) and zyg-2(b10ts) are alleles of mus-101

Mapping data of *emb-10* placed it on chromosome I at 5.74 cM and *zyg-2* was previously mapped to chromosome I at 6.24 cM. WGS/SNP mapping placed *emb-10* in a 0.9-Mb interval on chromosome I and *zyg-2* within a 5.2-Mb interval on chromosome I. Only two nonsynonymous sequence variants were identified for both *emb-10* (*mus-101* and *F59C6.8*, Table 4) and *zyg-2* (*mus-101* and *lrk-1*, Table 5). Previous studies had not tested whether these two mutants might be alleles of the same gene. However, the WGS/SNP mapping analysis found *mus-101* lesions as candidates for both mutants. In addition, *mus-101* was the only candidate reported to be embryonic lethal by RNAi. To validate whether the *mus-101* lesions were responsible for the *emb-10(k12ts)* and *zyg-2(b10ts)* phenotypes, we performed RNAi phenocopy experiments. However, *mus-101* RNAi was not embryonic lethal in our hands.

**Table 4:** *emb-10(k12ts)* is an allele of *mus-101*

Strain	Candidate	Mutation	Description	RNAi Phenocopy—Reported on Wormbase	RNAi Phenocopy—This Study	Complementation Test
*emb-10(k12ts)*	***mus-101***	G->A, Cys268Tyr	Chromosomal protein for DNA metabolic processes	Range of embryonic lethal	Not embryonic lethal	Fails to complement *mus-101(tm1761∆)* at 24° (1% hatching, N = 12)
	*F59C6.8*	C->T, Thr446Met	No information available	Not embryonic lethal	Not embryonic lethal	NP

List of genes with genomic DNA sequence variants identified by WGS/SNP mapping of *emb-10(k12ts)*, the missense mutation identified, and corresponding amino acid change, a description of the gene function, RNAi phenotypes reported on Wormbase, the percent embryonic lethality by RNAi experiments performed in this study, and the results of complementation tests performed in this study. NP, not performed. N is the number of F1 *trans*-heterozygous animals shifted to 24° for F2 hatching analysis. RNAi, RNA interference; WGS, whole-genome sequencing; SNP, single-nucleotide polymorphism. Bold indicates the gene, when mutated, that is responsible for the embryonic lethal phenotype.

**Table 5:** *zyg-2(b10ts)* is an allele of *mus-101*

Strain	Candidate	Mutation	Description	RNAi Phenocopy—Reported on Wormbase	RNAi Phenocopy—This Study	Complementation Test
*zyg-2(b10ts)*	***mus-101***	G->A, Trp1062stop	Chromosomal protein for DNA metabolic processes	Range of embryonic lethal	Not embryonic lethal	Fails to complement *mus-101(tm1761∆)* at 24° (0% hatching, N = 9)
	*lrk-1*	G->A, Gly1244Asp	Leucine-rich repeat kinase	Not embryonic lethal	Not embryonic lethal	NP

List of genes with genomic DNA sequence variants identified by WGS/SNP mapping of *zyg-2(b10ts)*, the missense mutation identified, and corresponding amino acid change, a description of the gene function, RNAi phenotypes reported on Wormbase, the percent embryonic lethality by RNAi experiments performed in this study, and the results of complementation tests performed in this study. NP, not performed. N is the number of F1 *trans*-heterozygous animals shifted to 24° for F2 hatching analysis. RNAi, RNA interference; WGS, whole-genome sequencing; SNP, single-nucleotide polymorphism. Bold indicates the gene, when mutated, that is responsible for the embryonic lethal phenotype.

**Figure 5:**
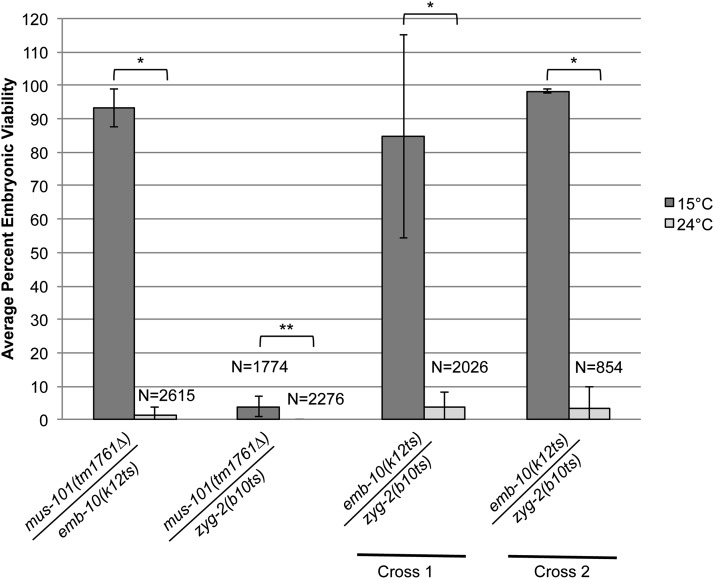
*emb-10(k12ts)* and *zyg-2(b10ts)* fail to complement *mus-101(tm1761∆)* and each other. The following complementation tests were performed: *emb-10(k12ts)* males crossed with *mus-101(tm1761∆)/hT2* hermaphrodites, *zyg-2(b10ts)*; *him-8(e1489)* males crossed with *mus-101(tm1761∆)/hT2* hermaphrodites, *emb-10(k12ts)* males crossed with *zyg-2(b10ts)* hermaphrodites (Cross 1), and *zyg-2(b10ts)*; *him-8(e1489)* males crossed with *emb-10(k12ts)* hermaphrodites (Cross 2). The progeny of single F1 *trans*-heterozygous hermaphrodites were scored for the number of dead embryos *vs.* larvae and the results calculated as the average percent embryonic viability at 15° or 24°. The number of *mus-101(tm1761∆)/emb-10(k12ts)* hermaphrodites scored at 15° = 12 and 24° = 12. The number of *mus-101(tm1761∆)/ zyg-2(b10ts)* hermaphrodites scored at 15° = 8 and 24° = 9. The number of *emb-10(k12ts)/zyg-2(b10ts)* hermaphrodites scored for Cross 1 at 15° = 10 and 24° = 12 and for Cross 2 at 15° = 3 and 24° = 4. N = total number of embryos and hatched larvae scored. *P < 0.0001. **P < 0.05. Error bars indicate standard deviation. At least 854 embryos were scored for each cross.

Mutant analysis had previously determined that *mus-101(tm1761∆)* was sterile and/or lethal. To validate whether the *mus-101* genetic lesions identified from WGS of *emb-10(k12ts)* and *zyg-2(b10ts)* were indeed alleles of *mus-101*, complementation tests were performed. Matings between *emb-10(k12ts)* males or *zyg-2(b10ts)*; *him-8* males and *mus-101(tm1761∆)/hT2* hermaphrodites were performed. Single F1 *trans*-heterozygous cross progeny were analyzed for the number of dead F2 embryos *vs.* hatched F2 progeny at 15° and 24°. *mus-101(tm1761∆)* failed to complement both *emb-10(k12ts)* and *zyg-2(b10ts)* (1% and 0% hatching at 24°, Table 4, and Table 5, and Figure 5) demonstrating that all three were allelic. In addition, embryonic viability of *mus-101(tm1761∆)/zyg-2(b10ts)* F1 *trans*-heterozygotes at the permissive temperature of 15° was only 3.9% (Figure 5) suggesting that the nonsense allele of *zyg-2(b10ts)* is stronger than the missense allele of *emb-10(k12ts)* when combined with the deletion allele of *mus-101(tm1761∆)*. To further validate that *emb-10(k12ts)* and *zyg-2(b10ts)* are two alleles of the same gene, we crossed *emb-10(k12ts)* males with *zyg-2(b10ts)* hermaphrodites and analyzed the progeny of single F1 *trans*-heterozygous animals. *emb-10(k12ts)* and *zyg-2(b10ts)* fail to complement with only 3.6% hatching at 24° (Figure 5, Cross 1). The reciprocal mating [*zyg-2(b10ts)*; *him-8* males crossed with *emb-10(k12ts)* hermaphrodites] also failed to complement (3.5% hatching, Cross 2). These data demonstrate that *emb-10(k12ts)* and *zyg-2(b10ts)* are temperature-sensitive alleles of *mus-101*, a member of the Mus101 gene family involved in DNA damage repair and cell-cycle checkpoint control. In *C. elegans* the *mus-101* homolog is a 1227 amino acid protein that plays a role in both DNA replication and in the response to DNA damage (Holway *et al.* 2005). *emb-10(k12ts)* is a G→A change resulting in Cys268 to a Tyr in one of the six BRCT domains, which mediate protein−protein and protein−DNA interactions. *zyg-2(b10ts)* is a G→A transition that changes Trp1062 to a premature stop codon. The 165-bp region removed by the *zyg-2(b10ts)* premature stop is not predicted to contain a functional domain. However, an oligomerization domain resides in the C-terminus of the human homolog TOPBP1 (Sokka *et al.* 2010).

### *mel-2(it20)* is an allele of *tofu-6*

*mel-2(it20)* was originally identified in a screen for maternal-effect lethal mutants on linkage group II and was mapped to the left of *dpy-10* (Kemphues *et al.* 1988). WGS/SNP mapping found a 1.3-Mb interval with a reduction in Hawaiian SNPs on chromosome II (Table 6). Seven homozygous, nonsynonymous sequence variants were identified within that interval; two variants were identified within the same gene, *T14B4.2*. Two of the candidates were not reported to have an embryonic lethal phenotype by RNAi (*C25H3.8* and *F41G3.20*), and one was reported to have a low penetrance of embryonic lethality (*cyp-4*) and were not investigated further (Table 6). The other three candidates were reported to have a range of embryonic lethality by RNAi (*tofu-6*, *C56C10.10*, and *T14B4.2*).

**Table 6:** *mel-2(it20)* is an allele of *tofu-6*

Strain	Candidate	Mutation	Description	RNAi Phenocopy—Reported on Wormbase	RNAi Phenocopy—This Study	Complementation Test
*mel-2(it20) unc-4(e120)*	***tofu-6***	C->T, Gln52stop	SRA stem-loop interacting RNA binding protein	Range of embryonic lethal	NP	*ytEx100* rescues embryonic lethality of *mel-2(it20)*. Two independent lines.
	*cyp-4*	C->T, Thr157Ile	Cyclophilin divergent member	Low-range embryonic lethal	NP	NP
	*C25H3.8*	G->A, Ser281Asn	No information available	Not embryonic lethal	NP	NP
	*C56C10.10*	G->A, Arg118Gln	No information available	Range of embryonic lethal	NP	NP
	*T14B4.2*	A->C, Asn94His	No information available	Range of embryonic lethal	NP	NP
	*T14B4.2*	A->C, Lys95Gln	No information available	Range of embryonic lethal	NP	NP
	*F41G3.20*	Insertion of a C between nucleotide 207 and 208; frameshift	No information available	Not embryonic lethal	NP	NP

List of genes with genomic DNA sequence variants identified by WGS/SNP mapping of *mel-2(it20) unc-4(e120)*, the missense mutation identified, and corresponding amino acid change, a description of the gene function, RNAi phenotypes reported on Wormbase, the percent embryonic lethality by RNAi experiments performed in this study, and the results of complementation tests performed in this study. NP, not performed. RNAi, RNA interference; WGS, whole-genome sequencing; SNP, single-nucleotide polymorphism. Bold indicates the gene, when mutated, that is responsible for the embryonic lethal phenotype.

Because *tofu-6(yt2)* was identified as an allele that caused 100% maternal-effect embryonic lethality in a screen for altered expression of an early embryonic reporter construct (Minasaki and Streit 2007), it was a likely candidate. A combination of classical mapping, SNP mapping, and sequencing identified the *yt2* allele as a premature stop codon. An extrachromosomal array (*ytEx100*) containing a 3245-bp genomic fragment including wild-type *tofu-6* rescued the embryonic lethality of *tofu-6(yt2)* (Minasaki and Streit 2007). To validate whether *mel-2(it20)* is an allele of *tofu-6*, we crossed *mel-2(it20) unc-4(e120)/++* heterozygous males with rolling *tofu-6(yt2)*; *ytEx100* [*ytEx100* is marked with a dominant marker *rol-6(su1006)*] hermaphrodites. The RolUnc progeny of F1 rollers were picked and scored for hatched progeny. All *mel-2(it20) unc-4(e120)*; *ytEx100* animals produced live progeny whereas Unc non-Rol animals that segregate from these lines exhibit a maternal-effect embryonic lethal phenotype (Table 6). These experiments demonstrate that a transgene carrying *tofu-6* can rescue *mel-2(it20)*. From these data we conclude that *mel-2(it20)* is an allele of *tofu-6*, a gene involved in cell division timing of the early embryo (Minasaki and Streit 2007). TOFU-6 encodes a 367 amino acid protein orthologous to SLIRP (human SRA stem-loop interacting RNA binding protein) with an RRM (RNA recognition motif) domain that spans amino acids 17−87. *mel-2(it20)* is a C→T transition that changes Gln52 to a stop codon. The premature stop truncates the protein at residue 52 disrupting the RRM domain.

### emb-14(g43ts) is an allele of fasn-1

*emb-14* was previously mapped to chromosome I at 1.71 cM. Analysis of the sequence data generated by WGS/SNP mapping of *emb-14(g43ts)* found a reduction of Hawaiian SNPs on chromosome I within a 2.2 Mb interval. This interval contained seven nonsynonymous sequence variants (Table 7). Of the seven candidates, only one was reported to have an embryonic lethal phenotype by RNAi (*fasn-1*) and one was reported as lethal or sterile by mutant analysis (*syp-4*). *fasn-1* RNAi phenocopied the embryonic lethal phenotype of *emb-14(g43ts)*, however, no deletion mutant was available for further validation by complementation tests. The deletion allele, *syp-4(tm2713∆)*, was used for complementation tests with *emb-14(g43ts)*. *syp-4(tm2713∆)/+* males were crossed with *emb-14(g43ts)* homozygous hermaphrodites and the progeny of *trans*-heterozygous F1 were examined for their ability to hatch at 15° and 24°. All F1 hermaphrodites produced viable progeny (81% hatching, Table 7). To further validate the identity of *emb-14(g43ts)* seven Dpy non-Unc and seven Unc non-Dpy recombinants were isolated from *emb-14(g43ts)/dpy-5unc-13* heterozygotes (Supporting Information, Figure S1A). After generating homozygous recombinants for the *dpy-5* or *unc-13* markers, these recombinants were shifted to 24° to test for the presence of *emb-14(g43ts)*. Each line was then subjected to polymerase chain reaction and sequencing of the *fasn-1* and *syp-4* genes and the sequences analyzed for the mutations identified by WGS. All seven Dpy non-Unc recombinants carried *emb-14(g43ts)* (*i.e.*, exhibited embryonic lethality at 24°). These seven lines also contained the *fasn-1* mutation (G→A, Ala1425 to Thr) and one of the lines was wild type for *syp-4* (Figure S1B). None of the seven Unc non-Dpy were embryonic lethal at 24°, suggesting that they did not contain *emb-14(g43ts)*. In addition, one of the Unc non-Dpy (not embryonic lethal) carried the *syp-4* mutation (Figure S1B). These genetic data combined with the sequencing data supports the conclusion that *emb-14* is allelic with the *fasn-1* gene. *fasn-1* is the *C. elegans* fatty acid synthase, which catalyzes the conversion of malonyl CoA into palmitate in the fatty acid biosynthetic pathway. Depletion of maternal *fasn-1* by RNAi causes a loss of anterior-posterior polarity in the progeny and osmotic sensitivity (Rappleye *et al.* 2003). A previously identified allele, *fasn-1(fr8)*, also has been found to play a role in the innate immune response (Lee *et al.* 2010). *The emb-14(g43ts)* mutation changes Ala1425 to Thr in a conserved beta-ketoacyl reductase domain, which is needed for reduction of the growing fatty acid chain.

**Table 7:** *emb-14(g43ts)* is an allele of *fasn-1*

Strain	Candidate	Mutation	Description	RNAi Phenocopy- Reported on Wormbase	RNAi Phenocopy- This Study	Complementation Test
*emb-14(g43ts)*	*pqn-20*	C->T, Ser51Phe	Prion-like-(Q/N-rich)-domain-bearing protein	Not embryonic lethal	NP	NP
	*syp-4*	C->T, Thr354Ile	Synapsis in meiosis abnormal	Not embryonic lethal	NP	Complements *syp-4(tm2713∆)* at 24° (81% hatching, N = 9)
	*T21G5.1*	C->A, Pro144Gln	No information available	Not embryonic lethal	Not embryonic lethal	NP
	*smg-1*	G->A, Trp652stop	Phosphatidylinositol kinase-related protein kinase	Not embryonic lethal	Not embryonic lethal	NP
	*lrp-1*	C->T, Pro645Leu	low-density lipoprotein (LDL) receptor-like protein	Not embryonic lethal	NP	NP
	*F26A3.1*	G->A, Glu276Lys	No information available	Not embryonic lethal	NP	NP
	***fasn-1***	G->A, Ala1425Thr	Fatty acid synthase	100% embryonic lethal	100% embryonic lethal	See text for genetic analysis of recombinants

List of genes with genomic DNA sequence variants identified by WGS/SNP mapping of *emb-14(g43ts)*, the missense mutation identified and corresponding amino acid change, a description of the gene function, RNAi phenotypes reported on Wormbase, the percent embryonic lethality by RNAi experiments performed in this study, and the results of complementation tests performed in this study. NP, not performed. N is the number of F1 *trans*-heterozygous animals shifted to 24° for F2 hatching analysis. RNAi, RNA interference; WGS, whole-genome sequencing; SNP, single-nucleotide polymorphism. Bold indicates the gene, when mutated, that is responsible for the embryonic lethal phenotype.

### *emb-29(g52ts)* is an allele of *cdc-25.2* and is rescued by CRISPR gene editing

Previous characterization placed *emb-29* on chromosome V at −18.34 cM and another group previously implicated *emb-29(g52ts)* as a complex allele of *cdc-25.2* (Nair *et al.* 2013). Using a different method of WGS, Nair *et al.* (2013) found a Ser239 to Phe change in both *emb-29(g52ts)* and *emb-29(b262)*. However, no definitive proof such as rescue experiments has ever been reported. Using the method of WGS/SNP mapping, we found a reduction of Hawaiian SNPs in a 1.3-Mb interval on chromosome V, consistent with previous characterization. Within this interval there were two nonsynonymous sequence variants, *nas-32* (Ala77 to Thr) and *cdc-25.2* (Ser239 to Phe) (Table 8). Only *cdc-25.2* was reported to be embryonic lethal by RNAi, but we never observed embryonic lethality by RNAi feeding.

**Table 8:** *emb-29(g52ts)* is an allele of *cdc-25.2*

Strain	Candidate	Mutation	Description	RNAi Phenocopy- Reported on Wormbase	RNAi Phenocopy- This Study	Complementation Test
*emb-29(g52ts)*	*nas-32*	G->A, Ala77Thr	Astacin family zinc metalloprotease	Not embryonic lethal	NP	NP
	***cdc-25.2***	C->T, Ser239Phe	Cdc25 phosphatase protein family homolog	Embryonic lethal penetrance 40–90%	not embryonic lethal	*cdc-25.2(ok597∆)* complements embryonic lethality at 24° (78.6% hatching, N = 20), but 91% of F2s are sterile (N = 34)

List of genes with genomic DNA sequence variants identified by WGS/SNP mapping of *emb-29(g52ts)*, the missense mutation identified and corresponding amino acid change, a description of the gene function, RNAi phenotypes reported on Wormbase, the percent embryonic lethality by RNAi experiments performed in this study, and the results of complementation tests performed in this study. NP, not performed. N is the number of F1 *trans*-heterozygous animals shifted to 24°C for F2 hatching analysis and the number of F2 animals with observed sterility. RNAi, RNA interference; WGS, whole-genome sequencing; SNP, single-nucleotide polymorphism. Bold indicates the gene, when mutated, that is responsible for the embryonic lethal phenotype.

Further validation by complementation has proven difficult. A deletion allele of *cdc-25.2* is homozygous sterile (Kim *et al.* 2010). We performed complementation tests by crossing homozygous *emb-29(g52ts)* males to *cdc-25.2(ok597∆)/hT2* hermaphrodites and analyzed single non-green *trans*-heterozygous progeny for the number of dead F2 embryos *vs.* live F2 progeny. Similar to Nair *et al.* (2013), we found that at 24° only ∼21% of progeny from *emb-29(g52ts)/cdc-25.2(ok597∆)* F1s die as embryos but 91% of the surviving progeny are sterile (Table 8). These data suggest that *emb-29(g52ts)/cdc-25.2(ok597∆)* complements the embryonic lethal phenotype of *emb-29(g52ts)* but fails to complement the sterility of *cdc-25.2(ok597∆)*.

**Figure 6:**
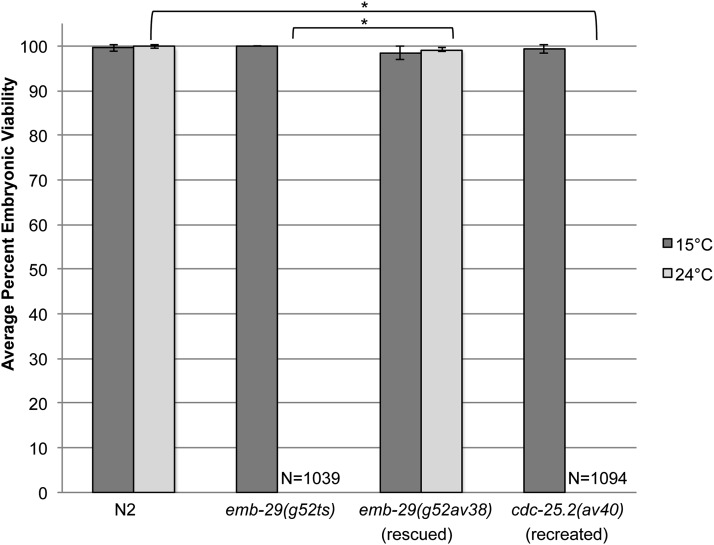
*emb-29(g52ts)* embryonic lethality is caused by a point mutation in *cdc-25.2*. Percent embryonic viability for control lines N2 (wild type) and *emb-29(g52ts)* and of converted lines *emb-29(g52ts)* to *emb-29(g52av38[T716C])*(rescued) and N2 to *cdc-25.2(av40[C716T])*(recreated). *P < 0.0001. Error bars indicate standard deviation. N = total number of embryos and hatched larvae scored. At least 860 embryos were scored for each genotype at each temperature.

Because there appears to be a complex genetic interaction between these two alleles making the complementation test results ambiguous, we rescued *emb-29(g52ts)* using the CRISPR/Cas9 gene editing system. *emb-29(g52ts)* was rescued by a single nucleotide conversion at nucleotide 716 of *cdc-25.2* with CRISPR/Cas9 gene editing and oligonucleotide-mediated recombination. We recovered three independent rescued lines. While embryonic viability of *emb-29(g52ts)* was 0% (1039 embryos) at 24°, the single-nucleotide change (T→C) led to an average of 99% hatching (2511 embryos) among the three rescued lines at 24°, similar to N2 (99.8% hatching; 1618 embryos, Figure 6). To further validate that this single nucleotide change in *cdc-25.2* is the cause of the *emb-29(g52)* temperature-sensitive embryonic lethal phenotype, we edited the genome of wild-type (N2) animals to recreate the *emb-29(g52ts)* allele. Although 99.8% (1618 embryos) of N2 progeny hatched at 24°, the percentage hatching of the recreated *emb-29(g52ts)* mutation (C→T) in three independent lines drastically decreased hatching to 0% (1094 embryos) at this temperature, similar to *emb-29(g52ts)* (0%; 1039 embryos, Figure 6). The rescue of *emb-29(g52ts)* and recreation of the temperature sensitivity by changing only a single nucleotide confirms that embryonic lethality in this line is in fact caused by the C716→T nucleotide change in *cdc-25.2*. *cdc-25.2* codes for one of four protein phosphatases (*cdc-25.1-cdc-25.4*) that promote cell cycle progression (Ashcroft *et al.* 1998). The Ser239 to Phe change of *emb-29(g52ts)* falls within a semi-conserved Rhodanese homology domain (amino acids 223-341). Members of the Rhodanese homology domain superfamily activate cell division kinases throughout the cell cycle.

## Discussion

Generating and characterizing an allelic series of mutants defective in a particular developmental process is a common strategy to understand how genes control that particular process. From classical work in phage and yeast, this approach has helped define complex biological processes from metabolic pathways to cell cycle division (Hartwell *et al.* 1974; Edgar *et al.* 1964). Pioneering work in the nematode, *C. elegans*, also applied a genetic approach to define nervous system development and behavior (Brenner 1973). In early attempts to understand the genetic control of embryonic development, many groups isolated thousands of mutants with an embryonic lethal phenotype (Hirsh and Vanderslice 1976; Vanderslice and Hirsh 1976; Miwa *et al.* 1980; Schierenberg *et al.* 1980; Wood *et al.* 1980; Cassada *et al.* 1981). Many of these legacy mutants have only been partially characterized and in many cases the causative molecular lesion remains unknown.

The traditional method for the identification of the phenotype-causing mutation is a long and laborious process. Mutagenized strains contain a substantial number of sequence variants. Even with the identification of sequence variants by WGS, it is often necessary to first map the mutation to a chromosomal interval via three-point mapping or a SNP mapping strategy. Both of these strategies require reiterative mapping to narrow down the relevant interval, which is a time-consuming process. We applied the WGS/SNP mapping strategy originally devised by Doitsidou *et al.* (2010) to identify the molecular lesions responsible for the embryonic lethal phenotype of mutants isolated 30 years ago. In this work, we have demonstrated that this is a relatively fast and efficient method to map and identify the molecular lesion of legacy mutants. The entire process, from the first outcross with Hawaiian males, to the picking of F2 progeny, making the library, and WGS can take place in 3−4 wk. The list of candidates can be generated in as little as 2 d. The complementation tests take about 7−10 d once all necessary strains have been obtained. The total protocol can be accomplished in less than 2 mo. We applied the WGS/SNP mapping strategy to seven legacy mutants and found that all seven of the mutants carried a single-point mutation resulting in a nonsynonymous amino acid change that caused the mutant phenotype, six of which were temperature-sensitive.

A recent paper used a non-SNP−based method of WGS to identify the molecular lesion of 64 mutants of essential genes on chromosome I (Chu *et al.* 2014). Although five of the mutants we identified had mutations in genes on chromosome I, none of these were identified in the work by Chu *et al.* (2014). In this work, the newly identified alleles corresponded to genes that have been studied by other groups ranging in processes from cell cycle regulators to components of the fatty-acid biosynthetic pathway. Many of the newly identified mutations are either in functional domains or in conserved regions of the corresponding protein. Because they are essential genes, mutations result in embryonic lethality precluding extensive characterization. With the identification of temperature-sensitive alleles, further studies are possible simply by changing the growth temperature.

In summary, we demonstrated that the WGS/SNP-based mapping strategy can be used to identify the unknown molecular lesions from the hundreds of legacy mutants that were isolated from early studies of *C. elegans* development, and that ts mutants are ideal targets for this approach. We have identified additional alleles of known genes and alleles of essential genes, which, previously, could only be studied by RNAi. All but one of these alleles is temperature-sensitive. We believe that these new temperature-sensitive alleles of essential genes are a valuable resource for the further investigation of essential developmental processes.
